# COMPARISON OF A FRAILTY RISK SCORE AND COMORBIDITY FOR EARLY REHOSPITALIZATION USING ELECTRONIC HEALTH RECORD DATA

**DOI:** 10.1093/geroni/igz038.3307

**Published:** 2019-11-08

**Authors:** Deborah A Lekan, Thomas P McCoy, Marjorie Jenkins, Somya Mohanty, Prashanti Manda

**Affiliations:** 1 UNC-Greensboro School of Nursing, Greensborol, North Carolina, United States; 2 University of North Carolina at Greensboro, Greensboro, North Carolina, United States; 3 Cone Health, Greensboro, North Carolina, United States; 4 University of North Carolina-Greensboro, Greensboro, North Carolina, United States

## Abstract

Frailty is a clinical syndrome of impaired homeostasis and decreased physiologic reserve and resilience resulting in diminished ability to recover from stressors. In the hospital setting, barriers to adoption of popular frailty assessments make them impractical for widespread use. Improving quality and costs associated with hospitalization has motivated using data from the electronic health record (EHR) to identify patients at risk for adverse outcomes such as early readmission. Patient-level factors such as frailty and comorbidity may signal high readmission risk. In this retrospective study and secondary analysis of EHR data, we investigated Frailty Risk Scores (FRS) in models that included sociodemographic, comorbidity, and laboratory data for early 3-, 7-, and 30-day unplanned readmission. Study data were collected from a health system in the Southeastern U.S. on adults >50 years with an inpatient stay of >24 hours, 2013-2017. Exclusions included planned readmission and in-hospital mortality. The FRS was constructed using ICD-10-CM codes mapped for symptoms, syndromes, and laboratory values. Cox and logistic regression were conducted to examine associations with readmission. Area under the receiver operating characteristic curve (AUC) quantified accuracy. The sample was 53% female and 73% non-Hispanic White (N=55,778). About one-third took at least 7 prescribed medications (34%) and average length of stay was 4.3 days (max=103.6). FRS was a significant predictor of readmission for almost all models, independently of three comorbidity indices (range AUC=.850-.854 for 3-day, .809-.813 for 7-day, and .757 to .768 for 30-day). Frailty and comorbidity are independently associated with early rehospitalization.

